# Cytologic Grading of Canine Mammary Tumors by Robinson's System Adapted to Romanowsky‐Type Rapid Staining: Prognostic Implications

**DOI:** 10.1111/vcp.70099

**Published:** 2026-02-26

**Authors:** Kaian F. Cavalcante, Francisco E. P. Cavalcante, Isabela R. B. Nascimento, Lúcia D. M. Silva, Augusto M. R. Faustino, Breno Q. Pinheiro, Isaac N. G. Silva

**Affiliations:** ^1^ Laboratório de Patologia Clínica Veterinária, Faculdade de Veterinária Universidade Estadual do Ceará Fortaleza CE Brazil; ^2^ Laboratório de Reprodução de Carnívoros, Faculdade de Veterinária Universidade Estadual do Ceará Fortaleza CE Brazil; ^3^ Departamento de Patologia e Imunologia Molecular, Instituto de Ciências Biomédicas de Abel Salazar Universidade do Porto Porto Portugal

**Keywords:** cytological grade, dog, mammary neoplasm, overall survival, prognosis, staining

## Abstract

**Background:**

Mammary neoplasms are a major concern in intact female dogs, and cytologic examination is routinely used for initial screening. Although the adaptation of Robinson's cytologic grading system to canine species has been scarcely explored, it shows prognostic potential, particularly when correlated with other clinical and pathologic markers.

**Objectives:**

This study aimed to assess the prognostic performance of an adaptation of Robinson's cytologic grading system using rapid Romanowsky‐type staining in canine mammary carcinomas. We also investigated its association with prognostic and predictive markers and overall survival.

**Methods:**

Fifty‐one cytologic samples from canine mammary tumors were analyzed to evaluate the association between cytologic grade, histopathology, Ki‐67 and Cox‐2 expression, clinical data, and survival. ROC curve analysis was performed to determine prognostic performance, and principal component analysis (PCA) was used to explore morphologic patterns.

**Results:**

Cytologic grade was significantly associated with tumor size, ulceration, and cellular features linked to malignancy but revealed low agreement with histologic grade and demonstrated no significant correlation with Ki‐67 and Cox‐2 expression. Higher cytologic grades and increased atypia were correlated with reduced survival. Robinson's cytologic score, Ki‐67 index, Peña's histologic grade, and ulceration were key variables associated with the biologic and morphologic heterogeneity of the tumors.

**Conclusion:**

The Robinson cytologic grading system, when adapted to rapid Romanowsky‐type staining, has prognostic relevance in canine mammary tumors. When interpreted alongside other clinical and pathologic features, it may contribute to early prognostic stratification and therapeutic planning. The use of larger cohorts in future studies is recommended for their validation.

## 1 Introduction

Mammary neoplasms represent a major concern in both human and veterinary oncology. In dogs, this category of tumors is frequently diagnosed in intact females, accounting for an incidence rate of up to 42% and a prevalence of up to 70% for highly aggressive lesions. In the human species, mammary tumors are associated with a high risk of clinical complications and a persistent increase in cancer‐related mortality [1, 2, 3]. Despite the notable phenotypic differences in lesion prevalence between species, comparative interspecies studies on canine and human mammary tumors are supported by several biological, clinical, pathologic, and molecular similarities. Furthermore, both species naturally develop spontaneous mammary neoplasms, unlike other experimental models [3, 4, 5]. In veterinary practice, cytologic examination is commonly used as part of the screening protocol for mammary lesions, aiming to distinguish between benign and malignant processes. This method is recognized for its practicality, low cost, and the wide range of clinically relevant information it provides in a short time, including the potential detection of tissue metastases [6, 7, 8, 9].

In human oncology, a cytologic grading system for mammary neoplasms developed by Robinson [10] has been standardized and shows a significant correlation with the Scarff‐Bloom‐Richardson histopathologic grading system [11], considered the gold standard for accurate diagnosis of human mammary lesions [5]. In veterinary medicine, a few studies have explored the potential adaptation of Robinson's cytologic grading system for canine cytologic samples. There is limited emphasis on its correlation with well‐established prognostic and predictive parameters in canine mammary oncology, as well as insufficient investigation of the variety of cytologic staining techniques and their clinicopathologic implications, with the Giemsa standard stain, a variant of Romanowsky‐type stains, being the only one applied experimentally in previous publications [5, 8, 12]. Romanowsky‐type stains offer high contrast between cellular components, allowing clearer visualization of intracellular and intranuclear details, particularly when compared with routine hematoxylin and eosin (H&E) staining. This property facilitates the identification of key morphologic criteria in cytologic evaluation of mammary lesions, especially when grading systems based on nuclear features, such as that proposed by Robinson [10], are applied [5, 13].

In this context, beyond conventional cytologic analysis, the investigation of molecular markers has significantly contributed to the prognostic stratification of canine mammary carcinomas (CMCs). The proliferative index (Ki‐67) is recognized as a major prognostic marker for canine mammary neoplasms, showing strong correlation with multiple parameters indicative of high tumor aggressiveness, such as tumor size, histologic grade, and regional lymph node metastasis [14, 15]. Similarly, the inflammatory marker (COX‐2) has demonstrated high prognostic potential due to evidence of its overexpression in CMCs [16, 17]. Thus, a cytologic grading system that shows association with well‐established clinicopathologic and immunohistochemical prognostic markers could serve as a reliable indicator of clinical progression in affected patients. It may also guide clinical and therapeutic decision‐making and offer predictive value for survival and recurrence [5, 8, 18].

In summary, the present study aims to evaluate a cytopathologic grading system adapted from the Robinson model using rapid Romanowsky‐type staining as a prognostic tool for CMCs. It further seeks to investigate the association of this system with key prognostic and predictive parameters in CMNs (including Ki‐67 proliferative index and Cox‐2 expression), to determine its association with overall survival in affected patients, and to develop a clinical decision‐making flowchart based on Robinson's cytopathological grade.

## 2 Materials and Methods

### 2.1 Ethics Statement

This study was approved by the Animal Experimentation Ethics Committee of the Ceará State University (UECE) (protocol no. 11690648/2021), and all dog owners who agreed to participate in the current study received proper orientation and signed a consent form.

### 2.2 Study Design and Animal Selection

A total of 84 female dogs with mammary tumors were initially screened prospectively for inclusion in this study, without predilection for breed, age, or weight. The inclusion criteria considered were: (I) complete clinical history; (II) therapeutic protocol restricted to mastectomy procedures; (III) fine‐needle aspiration (FNA) specimens obtained immediately after surgery; (IV) subsequent histopathologic examination; (V) immunostaining for Ki‐67 and Cox‐2; and (VI) patient follow‐up. However, 33 individuals were excluded due to incomplete clinical records, previous oncologic treatments, and non‐existent or deteriorated histological samples, occurrences that may bias or even make impossible the experimentation process to achieve the objectives of this study, resulting in a final cohort of 51 dogs that met all inclusion criteria. The selected dogs were divided into five groups (stages) classified based on histopathologic diagnosis and clinical staging.

The tumor‐bearing dogs were subdivided as follows: (B) dogs with histopathologically confirmed only benign mammary tumors; (I) dogs with malignant mammary carcinomas measuring ≤ 3 cm in diameter, with no evidence of lymph node or distant metastasis; (II) dogs with malignant mammary carcinomas measuring 3–5 cm in diameter, without lymph node or distant metastasis; (III) dogs with malignant mammary carcinomas measuring > 5 cm in diameter, without evidence of metastasis; (IV) dogs with malignant mammary carcinomas of any size with confirmed regional lymph node metastasis.

The classification followed the TNM system and the guidelines of the World Health Organization (WHO) [19].

### 2.3 Clinical Evaluation and Surgical Removal

Each dog underwent a complete clinical examination, including tumor measurement, lymph node palpation, and assessment for ulceration or necrosis. Blood samples were collected for hematologic and biochemical analysis. Thoracic radiographs (three projections) and abdominal total ultrasound were performed to evaluate metastatic spread.

All dogs underwent early surgical therapy, with a variety of procedures established based on the size, fixation to the surrounding tissue, and number of lesions when removing both tumors from the mammary gland and regional lymph nodes (inguinal and/or axillary) [9].

### 2.4 Cytologic Evaluation

Cytologic samples of the tumors were obtained after surgical excision using FNA. Tumors with a diameter greater than 3 cm were subdivided into four quadrants, each subjected to a separate sampling procedure [20]. Microscope slides were prepared using either the slide‐over‐slide smear (squash) or interrupted smear techniques, followed by air drying and rapid Romanowsky‐type staining [21].

Slides containing high‐quality diagnostic material were first examined under low magnification (40×) to identify areas with the highest concentration of well‐preserved, well‐stained, and evenly distributed cells. Cellularity, distribution, and spatial arrangement were assessed under intermediate magnification (100×). Cell types, subcellular details, and background elements were evaluated under high magnification (400× and 1000× using immersion oil), with a minimum of 30 fields examined. Blinding was applied to ensure that the cytologic interpretations were not influenced by preexisting clinical or histopathologic knowledge, thereby enhancing the objectivity and reproducibility of the cytologic grading process.

Cytopathologic classification was performed according to the criteria described by Dolka et al. [8] and Emanuelli et al. [1] Cytologic grading was determined using a modified version of the Robinson system, similar to the approach described by Dolka et al. [8], based on the following parameters: cell dissociation, nuclear size, cellular uniformity, nucleoli, nuclear margins, and chromatin pattern [8, 10]. The main methodologic distinction lies in the staining technique, as this study employed a rapid Romanowsky‐type stain instead of the standard Giemsa, since the former requires less time to perform and is more commonly used in routine laboratory services [13, 21].

### 2.5 Histopathologic Evaluation

Samples of tumors and respective regional lymph nodes were fixed in 10% buffered formalin, embedded in paraffin, and cut in a microtome to a thickness of 4 μm. The sections were stained with H&E for histopathologic evaluation, performed according to the system developed by Zappulli et al. [22] The histologic grading was determined based on tubular formation, nuclear pleomorphism, and mitotic index [18].

### 2.6 Patient Follow‐Up

All animals included in this study underwent a structured follow‐up protocol to assess clinical outcomes and survival. The total follow‐up period extended from the day of mastectomy (D0) to the last recorded contact, with a maximum follow‐up of 30 months. The long‐term follow‐up was conducted through telephone interviews with owners, focusing on clinic status, potential recurrence of neoplastic lesions, and survival status. The date of last contact was recorded for all patients, and in cases of confirmed death, the date of death was registered to calculate survival time.

### 2.7 Immunohistochemistry

Immunohistochemical analysis was performed on 3‐μm thick sections obtained from formalin‐fixed, paraffin‐embedded tumor samples. For antigen retrieval, slides were incubated in a sodium citrate buffer, followed by the application of a peroxidase blocker to inhibit the activity of endogenous peroxidases. The sections were incubated overnight at 4°C with either monoclonal mouse anti‐Ki‐67 antibody or rabbit monoclonal anti‐COX‐2 antibody, and detection was carried out using the Novolink Max Polymer Detection System with DAB chromogen, followed by counterstaining with Mayer's hematoxylin.

The Ki‐67 index was determined by manual counting of 1000 tumor cells in hot‐spot areas at 400× magnification. The proliferative index was expressed as the percentage of positively stained nuclei. The optimal cutoff point of ≥ 20% was adopted, as recommended by Cassali et al. [9].

COX‐2 expression was evaluated semi‐quantitatively, considering both the proportion of stained cells and the intensity of cytoplasmic staining in five high‐power fields (400×). Distribution scores were defined as follows: 0 (absent), 1 (≤ 0%), 2 (11%–30%), 3 (31%–60%), and 4 (> 60%). Staining intensity was graded from 0 (negative) to 3 (strong). The final score, obtained by multiplying distribution and intensity scores (range 0–12), was categorized as low (0–5) or high [6, 7, 8, 9, 10, 11, 13], following the criteria described by Lavalle et al. [23] and adopted by Araújo et al. [14] Neoplasms, with a total score ≥ 6, were considered positive for significant expression of Cox‐2 [9].

### 2.8 Statistical Analysis

Statistical analyses were performed to assess associations between cytologic parameters (including Robinson's cytologic grading adapted for Romanowsky‐type rapid staining), histopathologic features, immunohistochemical markers (Ki‐67 and COX‐2), and clinical data in female dogs with mammary tumors. Normality and homoscedasticity were evaluated using the Shapiro–Wilk, Kolmogorov–Smirnov, and Levene's tests. Depending on data distribution, Student's *t*‐test, ANOVA, Mann–Whitney *U* test, and Kruskal–Wallis test were applied as appropriate. Correlations were estimated using Spearman's rank correlation coefficient. Kaplan–Meier survival curves with log‐rank tests were used to compare survival outcomes between groups. The prognostic performance of cytologic grading was assessed using receiver operating characteristic (ROC) curve analysis. Global morphologic patterns were explored through principal component analysis (PCA). All statistical analyses were conducted using R software (version 4.3.0) and Python (version 3.9), adopting a significance threshold of *p* < 0.05.

## 3 Results

### 3.1 Characterization of Canine Mammary Tumors Based on Romanowsky‐Type Rapid Staining, Adapted Robinson's Cytologic Grades

The study included 51 female dogs with CMNs; the average age of the dogs was 9.85 ± 3.43 years, with a predominance of elderly animals. Their weight ranged from 2.4 to 41 kg, with an average of 10.12 ± 7.35 kg. Regarding breed distribution, 51.85% of the dogs were mixed‐breed, followed by Pinscher (14.81%), Poodle (9.26%), and Yorkshire Terrier (5.56%). Most of the animals were intact (81.48%), while 18.52% were spayed.

Among the cases of CMNs, 17.64% (9/51) were histologically classified as benign, while 82.35% (42/51) were malignant. Stage II and III were the most prevalent, representing 23.52% (12/54) of the cases each, followed by stages I (20.37%) and IV (15.68%).

Regarding the histopathologic grade (Table 1), the results indicated a statistically significant difference between the clinical staging groups (*p* = 0.0102), suggesting that tumors in more advanced stages present higher histologic grades. The post hoc test revealed that this difference was more evident between the extreme stages of tumor progression, with significant differences between stages I and IV (*p* = 0.0076) and stages II and IV (*p* = 0.0426).

**TABLE 1:** Clinical, histopathologic, and inflammatory indices in female dogs with CMNs, according to study groups.

	B	I	II	III	IV
Most frequent breed	Pinscher (33.33%)	Mixed‐breed (54.5%)	Mixed‐breed (53.8%)	Mixed‐breed (58.3%)	Mixed‐breed (62.5%)
Age (years)	8.4 ± 3.9	11.5 ± 4.3	8.8 ± 3.0	9.3 ± 2.6	11.4 ± 2.3
Weight (kg)	9.4 ± 9.7	7.7 ± 4.1	9.2 ± 5.3	14.3 ± 10.5	9.4 ± 3.9
Reproductive status	Intact (100%)	Intact (63.6%)	Intact (100%)	Intact (83.3%)	Intact (75.0%)
Number of nodules	1.2 ± 0.4	2.3 ± 1.1	1.8 ± 1.3	2.3 ± 1.0	3.1 ± 2.3
Main location	Inguinal (55.5%)	Cranial abdominal (36.3%)	Inguinal (38.5%)	Inguinal (41.7%)	Inguinal (50.0%)
Most affected mammary gland	M5L (44.4%)	M3R (27.3%)	M5R (23.0%)	M5L (25.0%)	M5R (37.5%)
Nodule diameter (cm)	1.4 ± 1.5	1.3 ± 0.7	2.7 ± 0.9	5.1 ± 3.2	7.5 ± 5.3
Most frequent diagnosis	Complex adenoma (33.33%)	Complex carcinoma (54.5%)	Carcinoma in a mixed tumor (38.4%)	Complex carcinoma (25.0%)	Comedocarcinoma (25.0%)
Histopathologic grade	*	1.2 ± 0.4^a^	1.4 ± 0.5 ^a^	1.8 ± 0.9^b^	2.2 ± 0.7^b^

*Note:* The table presents the distribution of clinical and histopathologic variables in female dogs with CMNs, stratified into benign neoplasms (B) and carcinomas according to clinical staging (I, II, III, and IV). Left inguinal mammary gland (M5L) and right cranial abdominal mammary gland (M3R). Numerical values are presented as mean ± standard deviation, and relative frequencies are expressed as percentages (%).

* Not applicable to this subgroup of the population. Superscript letters within the same row indicate statistically significant differences.

**FIGURE 1:**
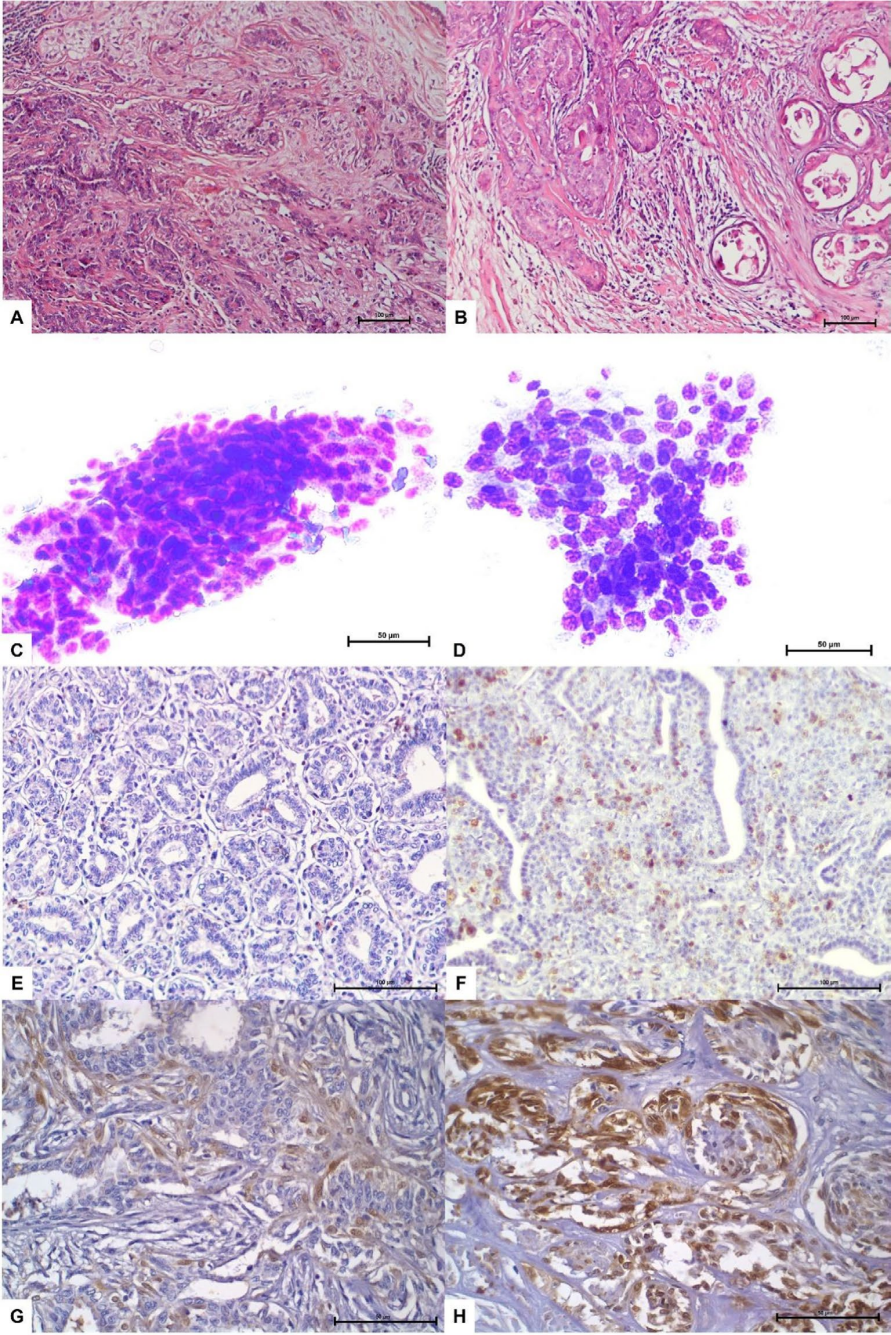
Histologic, cytologic, and immunohistochemical features of representative cases of canine mammary tumors. The panel includes histologic and cytologic subtypes (A–D) and immunohistochemical expression patterns of Ki‐67 and COX‐2 (E–H). (A) Complex adenoma (H&E, ×10 objective); (B) complex carcinoma (H&E, ×10 objective); (C) cytologic sample from complex adenoma showing a uniform cluster of monomorphic epithelial cells (Romanowsky‐type staining, ×40 objective); (D) cytologic sample from solid carcinoma showing pleomorphic epithelial cells in a poorly cohesive cluster, with nuclear pleomorphism, molding, coarse chromatin, and prominent nucleoli (Romanowsky‐type staining, ×40 objective); (E) fibroadenoma with low Ki‐67 expression (IHC, ×20 objective); (F) ductal carcinoma with high Ki‐67 expression (IHC, ×20 objective); (G) micropapillary carcinoma with low COX‐2 expression (IHC, ×40 objective); and (H) carcinoma in mixed tumor with high COX‐2 expression (IHC, ×40 objective). Scale bars are indicated in each panel.

Cytologically, 23.5% (12/51) of the tumors were classified as benign, while 76.5% (39/51) were malignant. Regarding malignant tumors, Robinson grades II and III were the most prevalent, each accounting for 43.5% (17/39), followed by grade I, with 13% (5/39). Clinical and pathologic features of canine mammary tumors according to Robinson's cytologic grading are summarized in Table 2. In general, the mammary gland M5L and complex carcinomas were the most frequently observed across cytologic grades (Figure 1A–D). Early clinical stages predominated among malignant cases. Tumors associated with higher cytologic grades tended to present larger mean diameters, while the number of nodules remained relatively stable among groups. Ulceration was infrequent overall, whereas necrosis controversy showed a higher occurrence in tumors classified with lower cytologic grades.

**TABLE 2:** Clinical and pathologic characteristics of canine mammary tumors according to Robinson's cytologic grade adapted to Romanowsky‐type rapid staining.

Robinson grade	Most affected mammary gland	Main histopathologic diagnosis	Main clinical stage	Number of nodules (mean ± SD)	Tumor diameter (mean ± SD)	Ulceration (n/N, %)	Necrosis (n/N, %)
0	M5L (33%)^A^	Carcinoma in mixed tumor (33%)^A^	III (42%)^A^	1.9 ± 0.9^A^	6.6 ± 4.9^A^	1/12 (8%)^A^	6/12 (50%)^A^
I	M3R (33%)^A^	Complex carcinoma (33%)^A^	II (33%)^A^	2.7 ± 0.6^A^	2.1 ± 1.4^AB^	0/5 (0%)^A^	2/5 (40%)^A^
II	M5L (29%)^A^	Carcinoma in mixed tumor (35%)^A^	I (35%)^A^	2.0 ± 1.8 ^A^	2.6 ± 2.6^B^	0/17 (0%)^A^	1/17 (6%)^A^
III	M4R (29%)^A^	Complex carcinoma (35%)^A^	III (29%)^A^	2.4 ± 1.3^A^	3.3 ± 2.5^AB^	3/17 (18%)^A^	1/17 (6%)^A^

*Note:* Most frequent mammary gland affected, main histopathologic diagnosis, and main clinical stage are expressed along with their relative frequency (%). Continuous variables are presented as mean ± standard deviation (SD). Ulceration and necrosis are presented as the number of positive cases per total cases within each grade (n/N), followed by the corresponding percentage. Robinson Grade 0 corresponds to benign lesions with no confirmed malignancy by cytology. Superscript uppercase letters within the same column indicate statistically significant differences (*p* < 0.05).

Histopathologic features of canine mammary tumors assessed by Peña's grading system, according to Robinson's cytologic grades, are presented in Table 3. Mean values of tubule formation, nuclear pleomorphism, and mitotic index were analyzed across cytologic grades, along with the distribution of the final histologic grade. Overall, tubule formation scores remained relatively low, while nuclear pleomorphism and mitotic activity showed mild variations among groups. The final histologic grade was predominantly low across all cytologic grades.

**TABLE 3:** Comparison between Romanowsky‐type rapid staining adapted Robinson's cytologic grading and Peña's histologic criteria in canine mammary tumors.

Robinson grade	Tubule formation (mean ± SD)	Nuclear pleomorphism (mean ± SD)	Mitotic index (mean ± SD)	Histologic grade (most frequent, %)
0	1.5 ± 1.0^A^	1.4 ± 0.8^A^	1.6 ± 1.1^A^	I (33%)^A^
I	2.3 ± 0.6^A^	2.0 ± 0.0^A^	2.3 ± 1.2^A^	III (67%)^A^
II	1.4 ± 0.8^A^	1.4 ± 0.8^A^	1.2 ± 0.8^A^	I (59%)^A^
III	1.6 ± 0.9^A^	1.7 ± 0.8^A^	1.2 ± 0.8^A^	I (47%)^A^

*Note:* Tubule formation, nuclear pleomorphism, and mitotic index are expressed as mean ± standard deviation (SD) according to Peña's histologic grading system. The final histologic grade is presented as the most frequent grade within each Robinson cytologic grade, accompanied by the corresponding percentage. Robinson Grade 0 corresponds to benign lesions with no confirmed malignancy by cytology. Superscript uppercase letters within the same column indicate statistically significant differences (*p* < 0.05).

Cytologic features used in Robinson's grading system, evaluated individually across cytologic grades, are summarized in Table 4. Mean values for cell dissociation, cell size, cell uniformity, nucleoli prominence, nuclear margin, and chromatin pattern were compared among groups. Overall, higher cytologic grades were associated with increased scores in all evaluated features, while lower grades exhibited lower scores across all parameters.

**TABLE 4:** Cytologic features according to Romanowsky‐type rapid staining adapted Robinson's grading system in canine mammary tumors.

Robinson grade	Cell dissociation (mean ± SD)	Nuclear size (mean ± SD)	Cell uniformity (mean ± SD)	Nucleoli (mean ± SD)	Nuclear margin (mean ± SD)	Chromatin (mean ± SD)
0	0.0 ± 0.0^A^	0.0 ± 0.0^A^	0.0 ± 0.0^A^	0.0 ± 0.0^A^	0.0 ± 0.0^A^	0.0 ± 0.0^A^
I	1.3 ± 0.6^B^	1.7 ± 0.6^B^	2.0 ± 0.0^B^	1.7 ± 0.6^B^	1.0 ± 0.0^B^	3.0 ± 0.0^B^
II	1.7 ± 0.5^bc^	2.0 ± 0.0^B^	2.1 ± 0.3^B^	2.6 ± 0.5^C^	1.8 ± 0.4^C^	2.9 ± 0.2^B^
III	2.1 ± 0.2^C^	2.1 ± 0.2^B^	2.9 ± 0.3^C^	3.0 ± 0.0^C^	2.3 ± 0.5^D^	3.0 ± 0.0^B^

*Note:* Cytologic criteria for Robinson's grading system, including cell dissociation, cell size, cell uniformity, nucleoli prominence, nuclear margin, and chromatin pattern, are presented as mean ± standard deviation (SD) for each Robinson cytologic grade. Robinson Grade 0 corresponds to benign lesions with no confirmed malignancy by cytology. Superscript uppercase letters within the same column indicate statistically significant differences (*p* < 0.05).

The immunohistochemical expression of Ki‐67 and Cox‐2 according to Robinson's cytologic grades is summarized in Table 5. No statistically significant differences were observed in the expression levels of Ki‐67 (*p* = 0.274) or Cox‐2 (*p* = 0.452) across the different cytologic grades, as determined by Kruskal–Wallis tests (Figure 1E–H).

**TABLE 5:** Ki‐67 and Cox‐2 expression according to Romanowsky‐type rapid staining adapted Robinson's cytologic grading in canine mammary tumors.

Robinson grade	Ki‐67 (%‐) (mean ± SD)	Cox‐2 (%) (mean ± SD)
0	19.79 ± 12.69^A^	0.89 ± 1.05^A^
I	37.27 ± 8.82^A^	1.00 ± 1.73^A^
II	16.90 ± 18.06^A^	2.12 ± 2.06^A^
III	22.24 ± 19.76^A^	1.71 ± 1.93^A^

*Note:* Expression levels of Ki‐67 (proliferation index) and Cox‐2 (inflammatory marker) are presented as mean ± standard deviation (SD) for each cytologic grade based on Robinson's classification. Values reflect immunohistochemical assessment of tumor samples. Superscript uppercase letters within the same column indicate statistically significant differences (*p* < 0.05).

### 3.2 Diagnostic Performance of Cytology and Romanowsky‐Type Rapid Staining Adapted Robinson's Grading System

**FIGURE 2:**
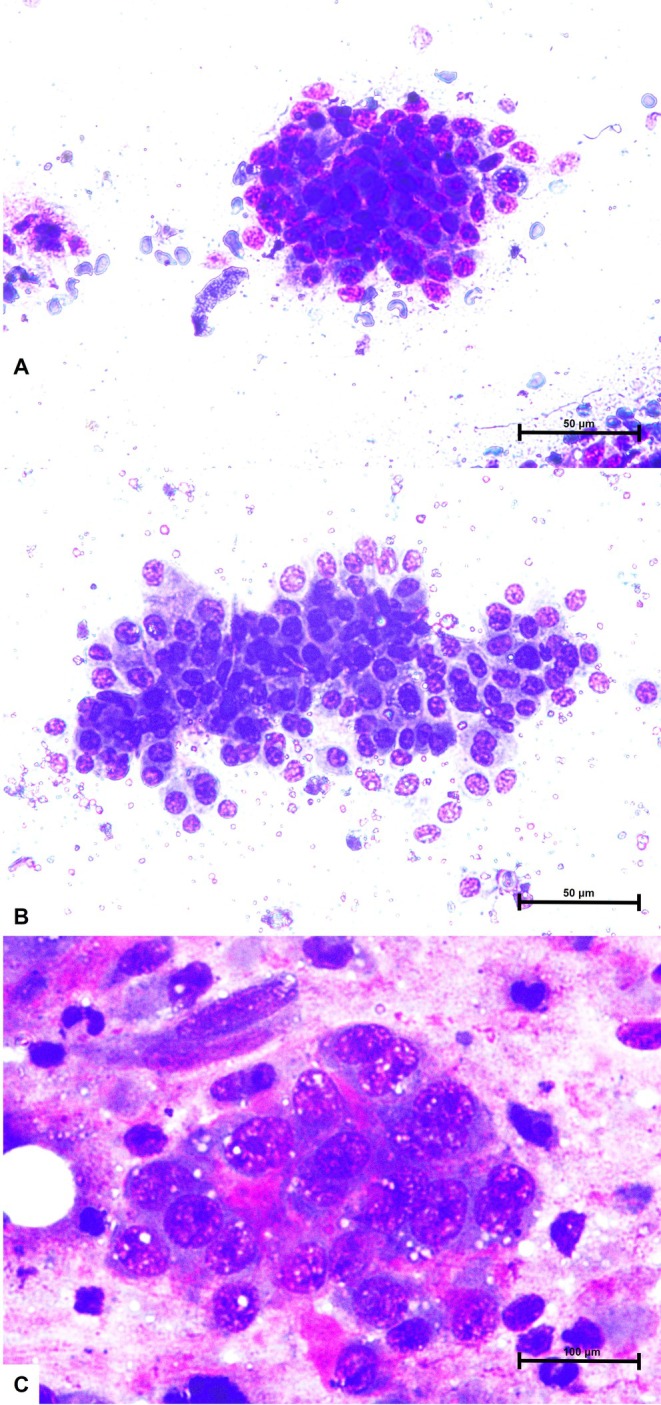
Cytologic features of canine mammary tumors according to Robinson's cytologic grading system. (A) Grade 1 mammary tumor showing cohesive clusters of epithelial cells with mild anisocytosis and anisokaryosis (Romanowsky‐type staining, ×40 objective). (B) Grade 2 mammary tumor exhibiting moderate cellular and nuclear pleomorphism, with irregular chromatin distribution, occasional prominent nucleoli and binucleation (Romanowsky‐type staining, ×40 objective). (C) Grade 2 mammary tumor at higher magnification demonstrating the cellular criteria influencing cytologic grading, including nuclear pleomorphism, anisocytosis, nucleoli, and chromatin pattern (Romanowsky‐type staining, ×100 objective). Scale bars are indicated in each panel.

**FIGURE 3:**
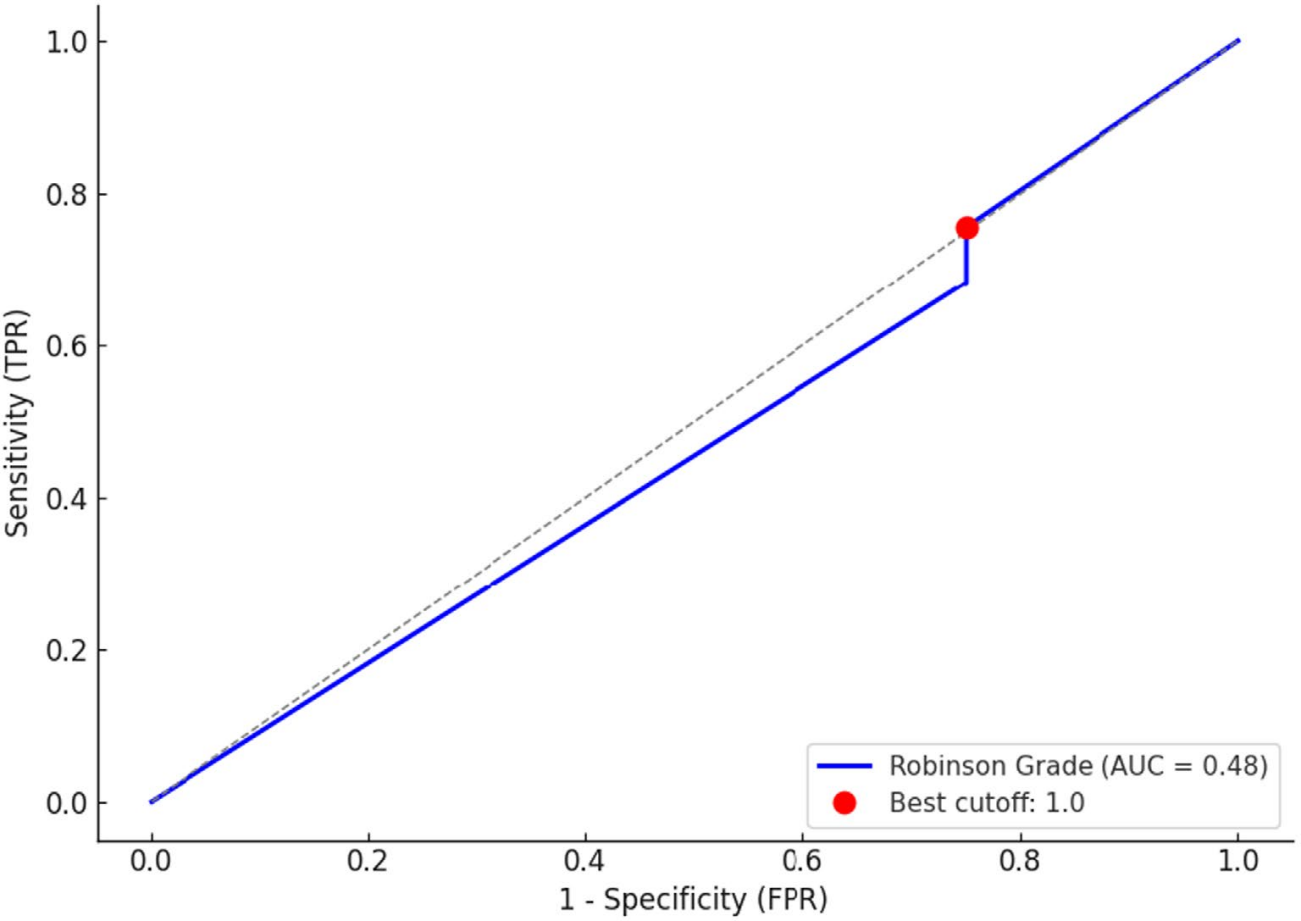
Receiver operating characteristic (ROC) curve for Robinson's cytologic grading in distinguishing benign from malignant canine mammary tumors.

To evaluate the sensitivity and specificity of cytology, low‐grade lesions according to Robinson's cytologic classification (grades 0) were considered benign, while grade one's lesions (grades 1, 2, and 3) were considered malignant (Figure 2). Histopathologic diagnosis was used as the gold standard for comparison. Of the 12 lesions cytologically classified as benign, 10 were histologically classified as malignant (false‐negative). Similarly, of the 39 tumors cytologically classified as malignant, 7 were histologically classified as benign (false‐positive). Based on this approach, cytology showed a sensitivity of 76.2%, a specificity of 22.2%, and an overall accuracy of 66.7% for distinguishing benign from malignant tumors. ROC curve analysis for Robinson's cytologic grade showed an AUC of 0.48, indicating a poor discriminative capacity between benign and malignant tumors. The optimal cutoff value was 1.0, yielding a sensitivity of 75.6% and a specificity of 25.0% for the detection of malignancy (Figure 3).

### 3.3 Impact of Robinson's Cytologic Grade Adapted to Romanowsky‐Type Rapid Staining on Survival Outcomes

Survival analysis based on Robinson's cytologic grading revealed a progressive decrease in survival rates across increasing cytologic grades.

**FIGURE 4:**
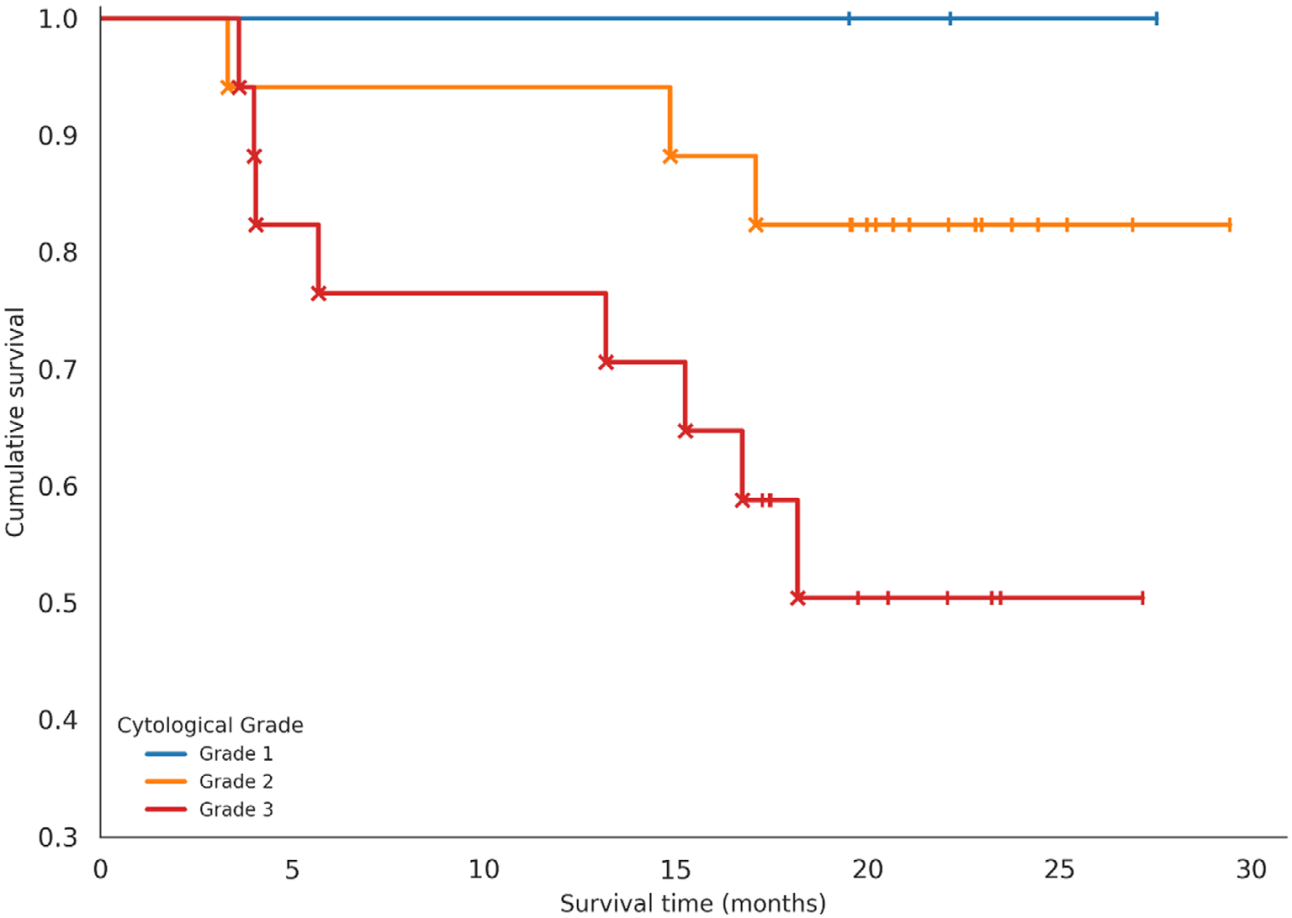
Kaplan–Meier survival curves of canine mammary tumors according to Robinson's cytologic grading.

No deaths were recorded among patients with grade 1 tumors (100% survival), while mortality rates reached 17.6% and 47.1% for grades 2 and 3, respectively. Although pairwise comparisons between grades did not reach statistical significance, the difference between grades 2 and 3 demonstrated a relevant trend (*p* = 0.0626) (Figure 4).

Moreover, Spearman's correlation analysis revealed a significant negative association between Robinson's cytologic grade and survival (ρ = −0.318; *p* = 0.0259), supporting the relationship between higher cytologic grades and poorer outcomes.

**FIGURE 5:**
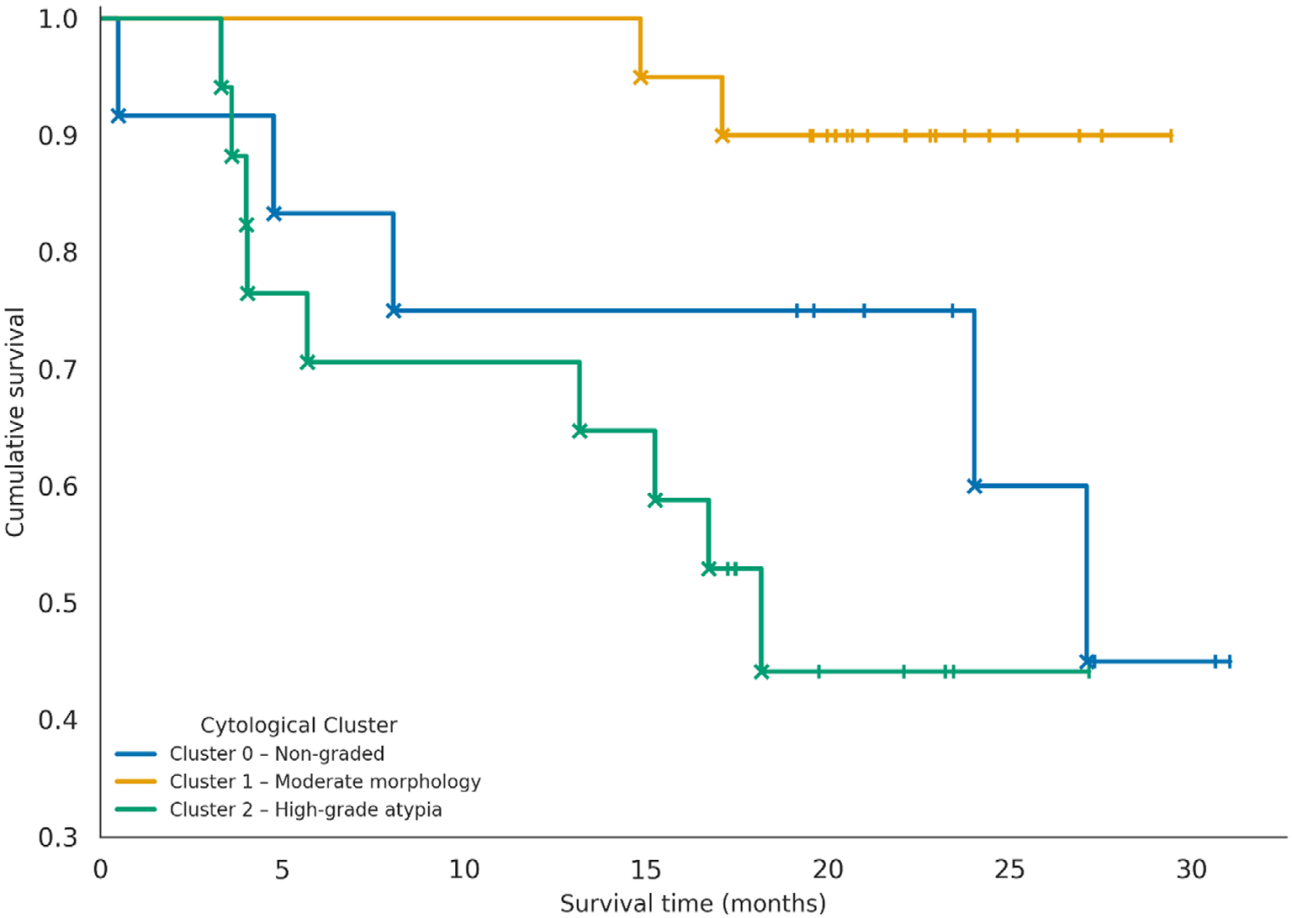
Kaplan–Meier survival curves of canine mammary tumors based on cytologic cluster analysis.

Despite the relatively small sample size, the hierarchical clustering analysis based on cytologic morphologic criteria identified three distinct clusters: Cluster 0 included non‐gradable samples, Cluster 1 encompassed cases with intermediate morphologic features, and Cluster 2 consisted of cases characterized by high cytologic atypia. Kaplan–Meier survival analysis revealed that Cluster 2 exhibited the poorest survival outcomes, with more than 50% mortality over the follow‐up period. In contrast, Cluster 1 showed a survival pattern similar to that observed in Robinson's grade 1 cases, while Cluster 0 (non‐gradable) demonstrated intermediate survival rates (Figure 5). In addition, a statistically significant difference in survival was observed between Clusters 1 and 2 (log‐rank test, *p* = 0.0024).

### 3.4 Exploration of Morphologic and Biological Patterns via Principal Component Analysis

**FIGURE 6:**
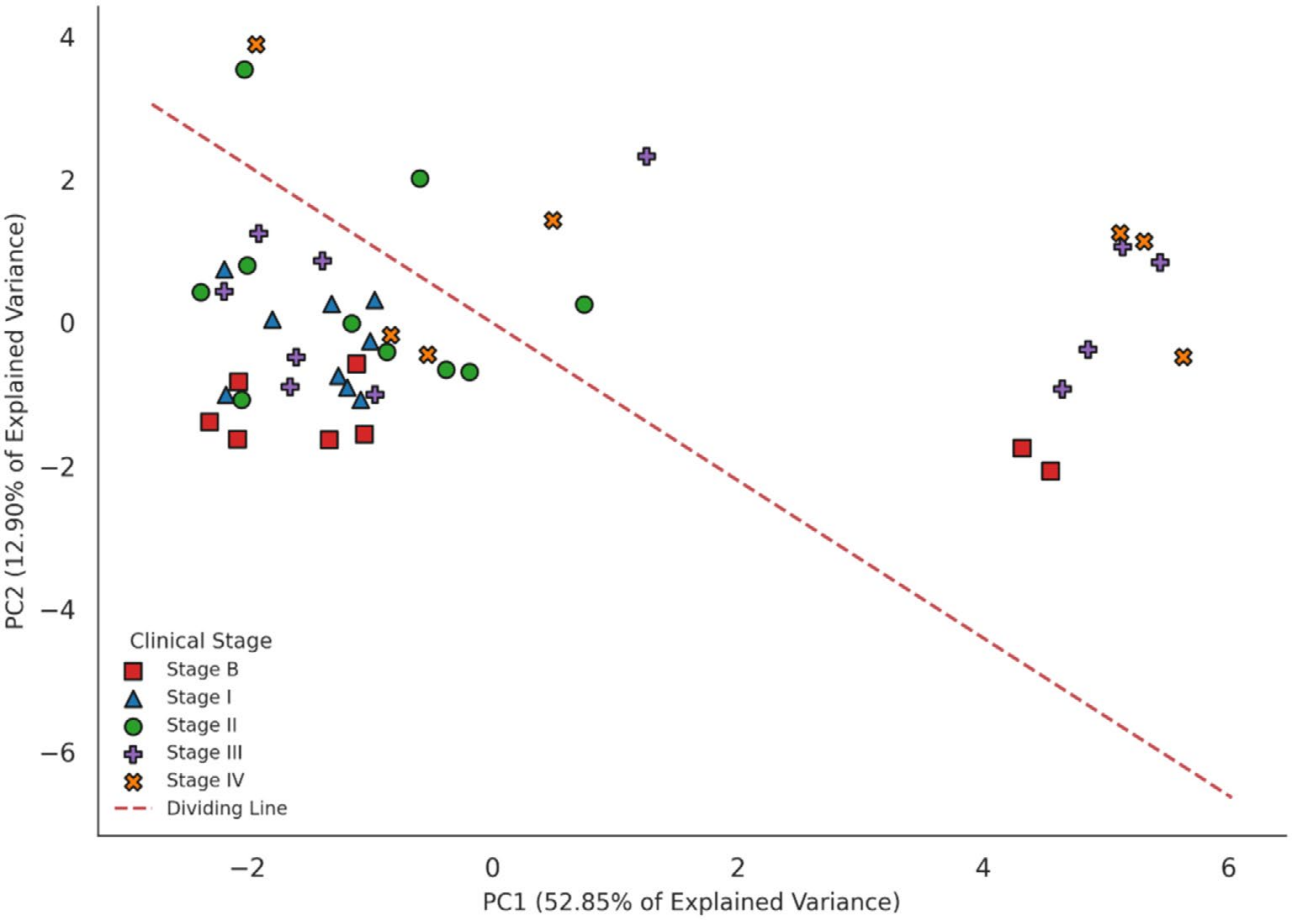
Principal component analysis (PCA) of canine mammary tumors based on cytologic and histopathologic features.

PCA identified two major biological axes explaining the morphologic and clinical heterogeneity of canine mammary tumors (Figure 6). The first principal component (PC1) accounted for 52.85% of the explained variance and primarily represented a cytomorphologic axis related to cellular atypia and tumor structural features. The most influential variables for PC1 were nuclear size (11.63%), Robinson's cytologic grade (11.60%), nucleoli prominence (11.58%), chromatin pattern (11.55%), and cell uniformity (11.41%). This component was strongly associated with classical morphologic features of malignancy and effectively synthesized the Robinson cytologic scoring system.

In contrast, the second principal component (PC2), which explained 12.90% of the variance, represented an alternative biological‐prognostic axis. PC2 was predominantly driven by Ki‐67 proliferation index (26.69%), Peña histologic grade (26.10%), tumor ulceration (18.86%), tumor necrosis (8.62%), and cell dissociation (4.15%). This component reflected a gradient of biological aggressiveness, highlighting characteristics related to rapid tumor growth, necrosis, and structural disorganization. Together, these findings suggest that cytologic morphology (PC1) and biological aggressiveness (PC2) are partially independent but complementary dimensions influencing tumor behavior and clinical outcomes, and indicate that Robinson's cytologic grading system may represent an important indicator of tumor progression and clinical prognosis of patients.

### 3.5 Proposed Preliminary Clinical Decision‐Making Algorithm Based on Robinson's Cytologic Grade Adapted to Romanowsky‐Type Rapid Staining

**FIGURE 7:**
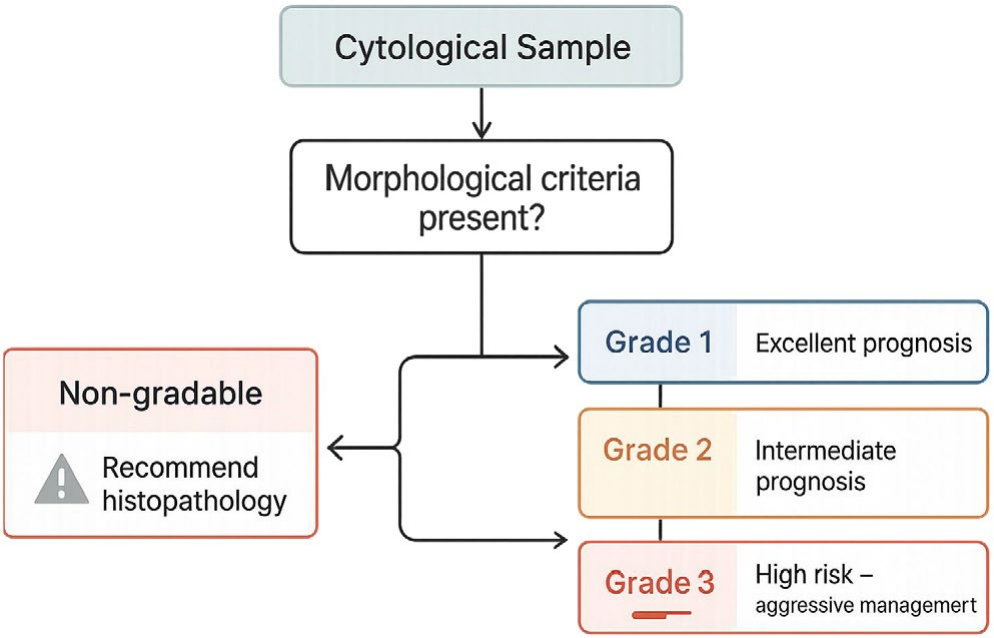
Proposed preliminary clinical decision‐making flowchart based on cytologic assessment and Robinson's grading of canine mammary tumors.

Based on statistical analysis and clinical outcomes, a preliminary interpretative clinical decision‐making flowchart was delineated for the management of canine mammary tumor cytologic samples assessed by Robinson's grading system adapted to Romanowsky‐type rapid staining (Figure 7).

Initially, samples were evaluated for the presence of sufficient morphologic criteria for scoring. Samples lacking adequate features (i.e., no criteria ≥ 1 point) were classified as non‐gradable (Cluster 0) and exhibited intermediate survival rates with notable mortality, underscoring the necessity for histopathologic confirmation even in the absence of cytologic grading.

For samples with sufficient cytologic criteria, Robinson's cytologic grade directed prognostic interpretation: (I) grade 1 samples demonstrated excellent prognosis, with 100% survival and no recorded deaths during follow‐up, suggesting that mild cytologic atypia corresponds to favorable outcomes when corroborated by clinical and imaging findings; (II) grade 2 samples represented an intermediate prognostic zone (17.6% mortality), without statistically significant differences compared with grade 3 (*p* = 0.0626). Management should integrate tumor size and the presence of ulceration, both of which significantly impact clinical outcomes; and (III) grade 3 samples exhibited the highest mortality rate (47.1%) and were consistently associated with tumor progression and probable metastasis, warranting aggressive staging investigations and expanded surgical interventions.

Additionally, cytologic features such as marked cell dissociation, visible nucleoli, nuclear irregularities, and dense chromatin, even when present in intermediate‐grade samples, were individually associated with adverse outcomes and should raise clinical suspicion.

This clinical model highlights that well‐interpreted cytology, combined with key clinical parameters, may serve not only as a screening tool for malignancy but also as a preliminary prognostic indicator, guiding initial therapeutic decisions more effectively.

## 4 Discussion

Cytology remains an accessible and practical diagnostic tool in veterinary oncology, particularly for distinguishing benign from malignant canine mammary neoplasms (CMNs) [5, 8]. Our findings confirm its high sensitivity and accuracy for detecting carcinomas, although specificity was markedly low. This aligns with previously reported patterns of false negatives [1, 24, 25, 26].

This study is one of the few to assess Robinson's cytologic grading system in CMNs using rapid Romanowsky‐type staining. Compared with the original Papanicolaou method [10], the rapid Romanowsky technique, which is consistent with the approaches adopted by Dolka et al. [8] and Soares [12], allowed enhanced visualization of nuclear features that were essential for grading. Key criteria such as nuclear margin, nucleoli prominence, and cell dissociation were directly associated with increasing cytologic grade, supporting previous observations [13].

Despite the difference in staining protocol, the cytologic grades identified in our study were generally consistent with previous veterinary and human studies, although we observed a higher frequency of grade III lesions [27, 28, 29]. This likely reflects the delayed clinical detection of CMNs in veterinary practice compared with human oncology [5, 30].

However, the concordance between cytologic and histologic grades was low. This may be due to the architectural limitations intrinsic to cytology, the morphologic complexity characteristic of CMNs, and the fact that some key histologic criteria are not accessible in cytologic analysis, such as tubular formations and even mitotic index [8, 18, 31]. Interestingly, most of the lesions classified as having high cytologic grades were attributed to histologic grade I, as shown in table 3, and this mismatch was also reported by previous authors [32, 33].

The inherent variability of CMNs, coupled with the more restricted cellular and structural context available to cytopathology, poses a challenge to accurate grading [5, 8, 34, 35]. Well‐differentiated malignant tumors may show minimal pleomorphism, mimicking benign patterns on cytology, while histologic progression can result in heterogeneous areas within the same lesion, with some demonstrating a benign appearance and others revealing marked malignancy, which can lead to misdiagnosis depending on the region from which the sample was obtained [8, 9, 25, 34, 35].

Robinson's system emphasizes nuclear features, such as nuclear margins, chromatin pattern, nucleoli, and size, while histologic grading focuses on tubular formation and mitotic index, which cannot be assessed cytologically [8, 10, 18, 24, 31]. The cytologic score, based on six parameters rated from one to three, reflects increasing cellular atypia and has been shown to correlate with overall survival [5, 8, 12]. In agreement with those findings, our study confirmed that higher cytologic grades, particularly grade III, could be associated with lower survival rates.

Several prognostic factors are frequently associated with CMN aggressiveness, including clinical staging, histologic grade, tumor size, number of nodules, ulceration, necrosis, and the expression of Ki‐67 and Cox‐2 [8, 9, 14, 18, 36, 37]. In our study, early clinical stages were more frequent in cytologically malignant tumors, and the number of nodules remained consistent across cytologic grades. Tumor diameter, however, was positively associated with cytologic grade, unlike findings in human studies [27]. Some cytologically benign tumors were large and clinically staged as advanced, likely reflecting misclassification.

Ulceration was more common in grade III lesions, as expected in more aggressive tumors, although it may also arise from ischemia or secondary infection [36, 37]. Necrosis did not correlate with cytologic grade, being more frequent in benign and grade I lesions, a pattern consistent with Yildirim and Gurel [38].

No significant correlation was found between cytologic grade and Ki‐67 or Cox‐2 expression. The distribution of Ki‐67 was particularly unexpected; with grade I lesions showing higher indices than grade III. This contradicts findings from previous studies in both species [2, 3, 14, 15, 39] but aligns with observations from Chauhan et al. [27] in a study of human mammary neoplasms. Such results may reflect the limited sample size, the prevalence of lower clinical stages between cytologic grades, and the inconsistencies between cytologic and histologic classifications.

Regarding Cox‐2, expression was uniformly low, supporting its variable significance as a prognostic marker, as it is naturally expressed by canine mammary tissue under normal conditions and non‐neoplastic pathologic conditions, such as inflammatory processes [2, 3, 16, 17, 40].

PCA indicates that Robinson's cytologic grade was the strongest factor associated with morphologic and clinical variability. It suggests that this was primarily driven by nuclear characteristics, confirming the high discriminative power of nuclear criteria in malignancy detection [21]. In addition, significant secondary contributions of histologic grade, Ki‐67, ulceration, necrosis, and cell dissociation were obtained, which collectively explained most of the data variation [8, 9, 14, 18, 36, 37].

The preliminary clinical decision‐making flowchart proposed here is a synthesis of the findings acquired in this study, aiming to support early risk stratification and guide surgical and therapeutic planning. Cytology, when interpreted alongside key clinical markers, could serve as an early prognostic tool, especially in settings where immediate histopathology is not available.

Nevertheless, the proposed model requires further studies to validate its accuracy before being applied, which should be done with caution and not as a substitute for histologic confirmation. The use of larger cohorts and improved standardization in cytologic interpretation is recommended as essential efforts to consolidate the role of cytologic grading in the prognostic assessment of CMNs.

This study reveals that the adaptation of the rapid Romanowsky stain to the Robinson classification demonstrates potential as a preliminary tool for malignancy screening and prognostic stratification in canine mammary tumors. The integration of cytologic interpretation with clinical parameters can increase its clinical utility and help in the understanding of complex prognostic evaluation, since morphologic patterns and biological aggressiveness emerged as distinct but complementary aspects in monitoring and understanding tumor behavior.

The proposed preliminary clinical decision‐making flowchart provides a structured approach for early decision‐making, emphasizing the importance of cautious interpretation even in unclassifiable cases. The findings of the present study support the use of cytology as an initial step in oncologic evaluation and encourage future studies to refine definitive cytologic prognostic models by incorporating molecular and histopathologic markers.

## Funding

This work was supported by Conselho Nacional de Desenvolvimento Científico e Tecnológico, #305420/2013‐5.

## Conflicts of Interest

The authors declare no conflicts of interest.
